# Supplementary material for: 14-3-3-protein regulates Nedd4-2 by modulating interactions between HECT and WW domains
Source: Commun Biol. 2021 Jul 22;4:899. doi: 10.1038/s42003-021-02419-0 (PMC8298602; doi:10.1038/s42003-021-02419-0)
Supplement: Supplementary file 1 — Supplementary Information [file 42003_2021_2419_MOESM1_ESM.pdf]

14-3-3-protein regulates Nedd4-2 by modulating interactions between HECT and WW domains

Pavel Pohl[1,2], Rohit Joshi[1,3], Olivia Petrvalska[1,3], Tomas Obsil[1,3*] and Veronika Obsilova[1*]

Affiliations

[1]Department of Structural Biology of Signaling Proteins, Division BIOCEV, Institute of Physiology of the Czech Academy of Sciences, 252 50 Vestec, Czech Republic

[2]2nd Faculty of Medicine, Charles University, V Uvalu 84, 15006 Prague, Czech Republic

[3]Department of Physical and Macromolecular Chemistry, Faculty of Science, Charles University, 128 43 Prague, Czech Republic

[*]**Corresponding authors:** veronika.obsilova@fgu.cas.cz (V.O.); obsil@natur.cuni.cz (T.O.)

Supplementary Information (available in this document)

Supplementary Figures S1–S6

Supplementary Tables S1–S5

Supplementary References

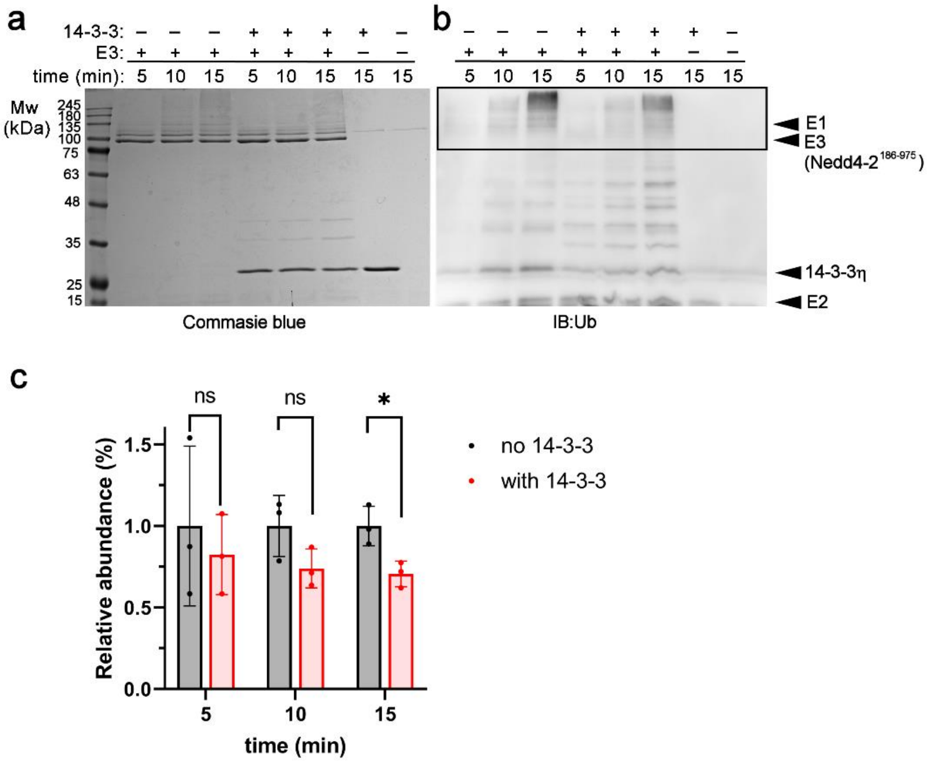

Supplementary Fig. S1. 14-3-3 slows down the auto-ubiquitination of Nedd4-2$^{186\text{-}975}$. a Purity and integrity of the samples used in the Ub chain formation assay. The samples were resolved by 10% Tris/Glycine SDS-PAGE and stained with Coomassie G-250. **b** Ubiquitin (Ub) chain formation assay with and without 14-3-3η and the representative immunoblot of Ub. Black rectangles denote the borders of the sample zones that were quantified. The presence of ubiquitinated Nedd4-2 in the quantified zones was confirmed by LC-MS analysis. **c** Relative abundances of ubiquitinated Nedd4-2$^{186\text{-}975}$ with and without 14-3-3η based on the immunoblot of Ub. Error bars represent the standard deviation of three independent experiments. Results show the data distribution. Asterisks represent significant differences according to unpaired Student's t-tests comparing relative change between samples without and with 14-3-3η at selected timepoints (ns, non-significant $P > 0.05$; *, $P < 0.05$).

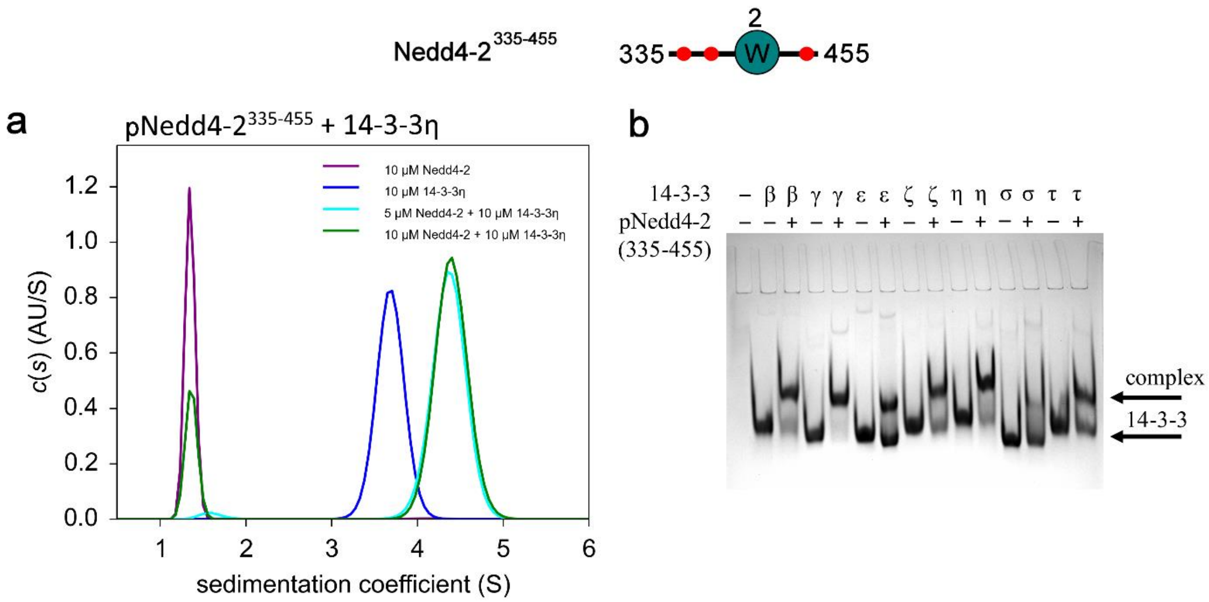

Supplementary Fig. S2. Characterization of the interaction between Nedd4-2$^{335-455}$ and 14-3-3 in solution. **a** Continuous sedimentation coefficient distributions (*c(s)*) of 10 μM pNedd4-2$^{335-455}$ alone (purple), 10 μM 14-3-3η alone (blue), and the pNedd4-2$^{335-455}$:14-3-3η complex mixed at 1:2 (cyan) and 1:1 (green) molar ratios. The protein concentrations were 10 μM for 14-3-3η and 5 μM or 10 μM for pNedd4-2$^{335-455}$. **b** Isoform binding specificity of pNedd4-2$^{335-455}$. Phosphorylated pNedd4-2$^{335-455}$, 14-3-3 proteins and their complexes were separated by 12% TBE-PAGE to study the interaction of pNedd4-2$^{335-455}$ with all seven human 14-3-3 isoforms (β, γ, ε, ζ, η, σ, and τ). The amounts of protein loaded on the native gel were 128 pmol of 14-3-3 and 64 pmol of pNedd4-2$^{335-455}$.

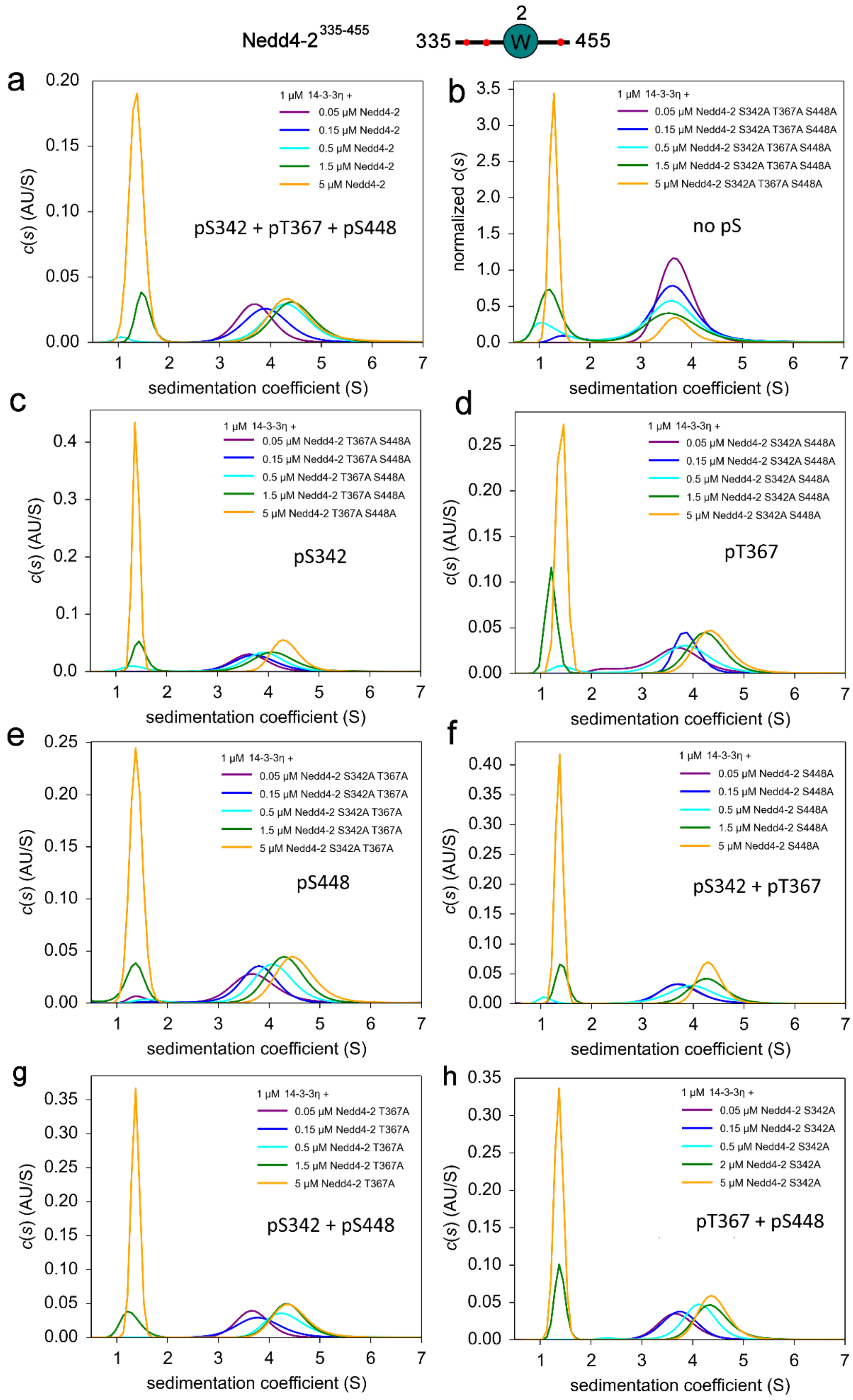
Nedd4-2 335-455
335
2
W
455
a
1 µM 14-3-3η +
0.05 µM Nedd4-2
0.15 µM Nedd4-2
0.5 µM Nedd4-2
1.5 µM Nedd4-2
5 µM Nedd4-2
pS342 + pT367 + pS448
c(s) (AU/S)
sedimentation coefficient (S)
b
1 µM 14-3-3η +
0.05 µM Nedd4-2 S342A T367A S448A
0.15 µM Nedd4-2 S342A T367A S448A
0.5 µM Nedd4-2 S342A T367A S448A
1.5 µM Nedd4-2 S342A T367A S448A
5 µM Nedd4-2 S342A T367A S448A
no pS
normalized c(s)
sedimentation coefficient (S)
c
1 µM 14-3-3η +
0.05 µM Nedd4-2 T367A S448A
0.15 µM Nedd4-2 T367A S448A
0.5 µM Nedd4-2 T367A S448A
1.5 µM Nedd4-2 T367A S448A
5 µM Nedd4-2 T367A S448A
pS342
c(s) (AU/S)
sedimentation coefficient (S)
d
1 µM 14-3-3η +
0.05 µM Nedd4-2 S342A S448A
0.15 µM Nedd4-2 S342A S448A
0.5 µM Nedd4-2 S342A S448A
1.5 µM Nedd4-2 S342A S448A
5 µM Nedd4-2 S342A S448A
pT367
c(s) (AU/S)
sedimentation coefficient (S)
e
1 µM 14-3-3η +
0.05 µM Nedd4-2 S342A T367A
0.15 µM Nedd4-2 S342A T367A
0.5 µM Nedd4-2 S342A T367A
1.5 µM Nedd4-2 S342A T367A
5 µM Nedd4-2 S342A T367A
pS448
c(s) (AU/S)
sedimentation coefficient (S)
f
1 µM 14-3-3η +
0.05 µM Nedd4-2 S448A
0.15 µM Nedd4-2 S448A
0.5 µM Nedd4-2 S448A
1.5 µM Nedd4-2 S448A
5 µM Nedd4-2 S448A
pS342 + pT367
c(s) (AU/S)
sedimentation coefficient (S)
g
1 µM 14-3-3η +
0.05 µM Nedd4-2 T367A
0.15 µM Nedd4-2 T367A
0.5 µM Nedd4-2 T367A
1.5 µM Nedd4-2 T367A
5 µM Nedd4-2 T367A
pS342 + pS448
c(s) (AU/S)
sedimentation coefficient (S)
h
1 µM 14-3-3η +
0.05 µM Nedd4-2 S342A
0.15 µM Nedd4-2 S342A
0.5 µM Nedd4-2 S342A
2 µM Nedd4-2 S342A
5 µM Nedd4-2 S342A
pT367 + pS448
c(s) (AU/S)
sedimentation coefficient (S)

Supplementary Fig. S3. Sedimentation velocity analytical ultracentrifugation analysis of the pNedd4-2$^{335\text{-}455}$:14-3-3η complexes corresponding to Fig. 3. The c(s) distributions of mixtures of 1 μM 14-3-3η with 0.05–5 μM pNedd4-2$^{335\text{-}455}$ variants: (**a**) pS342 + pT367 + pS448, (**b**) no phosphorylation site, (**c**) pS342, (**d**) pT367, (**e**) pS448, (**f**) pS342 + pT367, (**g**) pS342 + pS448 and (**h**) pT367 + pS448.

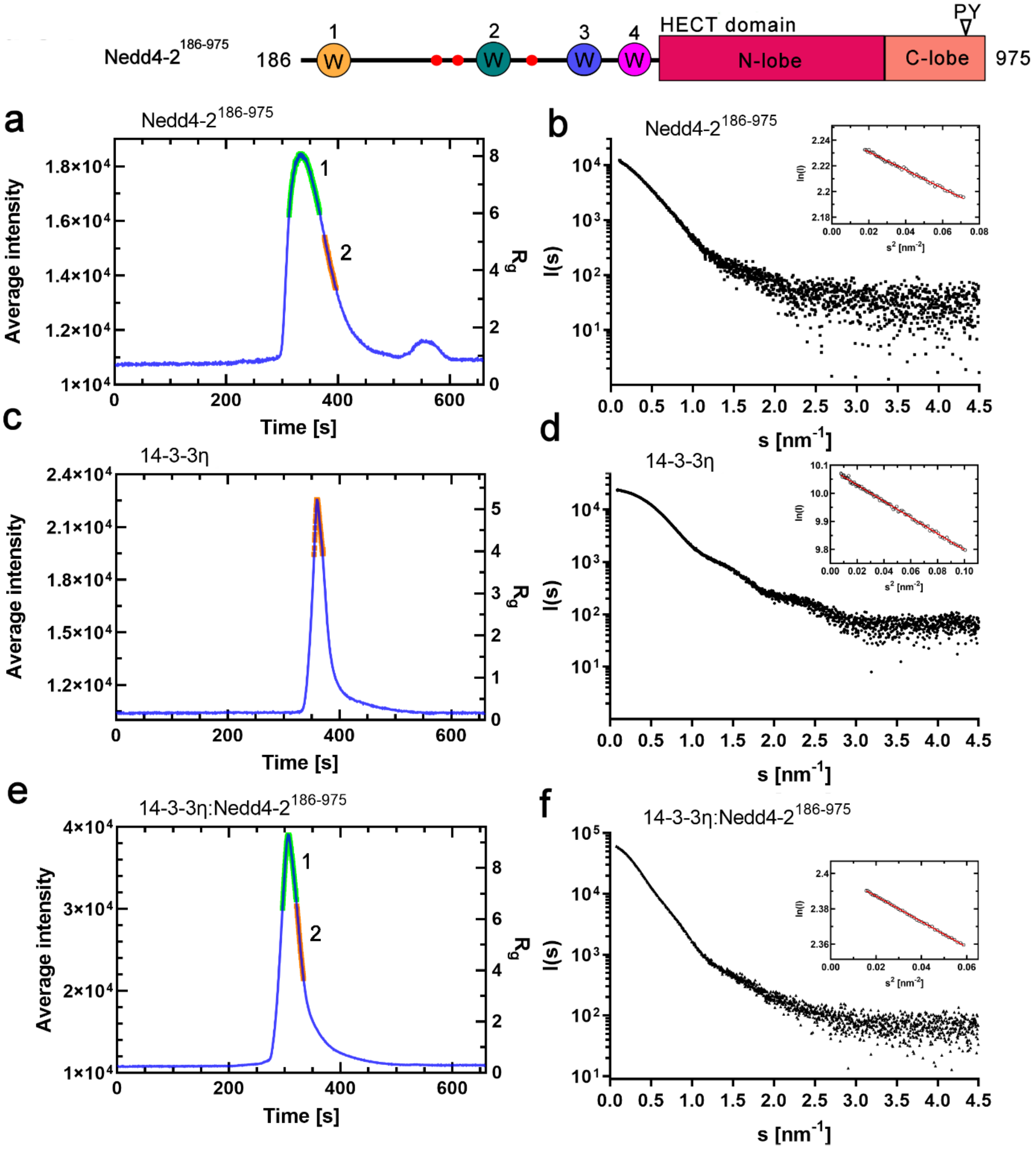

Supplementary Fig. S4. SEC-SAXS data analysis. Elution profiles of average intensity as a function of elution time (blue line) of (**a**) Nedd4-$2^{186\text{-}975}$ alone, (**c**) 14-3-3η and (**e**) 14-3-3η:pNedd4-$2^{186\text{-}975}$ complex eluting from the in-line SEC connected to SAXS instrumentation are shown on the left. Red lines denote the regions with the selected frames for final analysis, green lines (1) denote regions not selected for analysis. (**b,d,f**) The corresponding scattering curves from SEC-SAXS experiment are shown on the right. Scattering intensity *I(s)* is plotted in relation to the scattering vector s ($s = 4\pi\sin(\theta)/\lambda$, where 2θ is the scattering angle and λ is the wavelength). The insets are Guinier plots of low-*q* regions, with the red line showing linearity in ln(*I*) vs s^2.

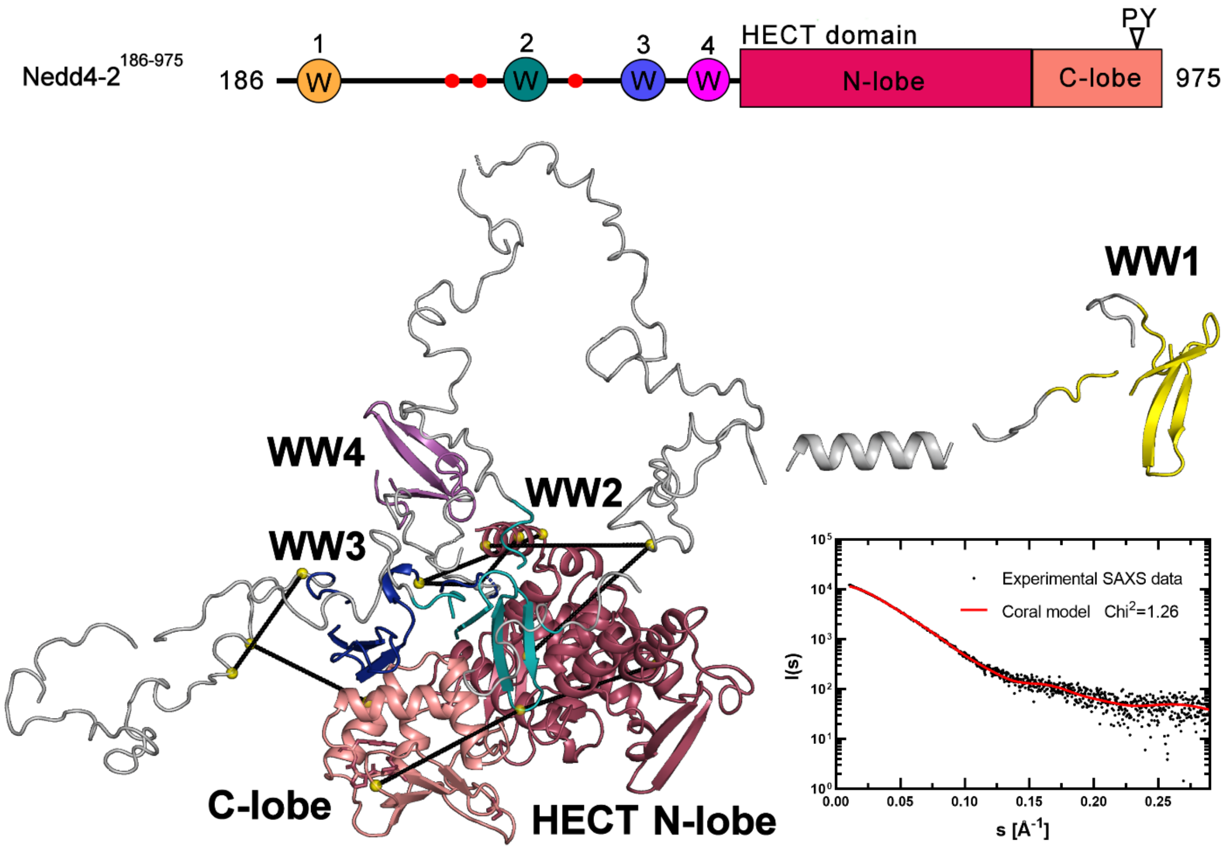

Supplementary Fig. S5: CORAL model of Nedd4-2$^{186\text{-}975}$ alone. Left, Best-scoring CORAL model of the Nedd4-2$^{186\text{-}975}$. HECT domain of Nedd4-2$^{186\text{-}975}$, N-lobe is shown in raspberry and C-lobe in salmon. WW1, WW2, WW3 and WW4 domains are shown in yellow, teal, blue and magenta, respectively. Flexible linkers between rigid bodies are shown in grey. Cα atoms of crosslinked residues are represented as yellow spheres. Right, scattering curve calculated from the CORAL model of Nedd4-2$^{186\text{-}975}$ (red) compared with the experimental SAXS data (black dots) [1].

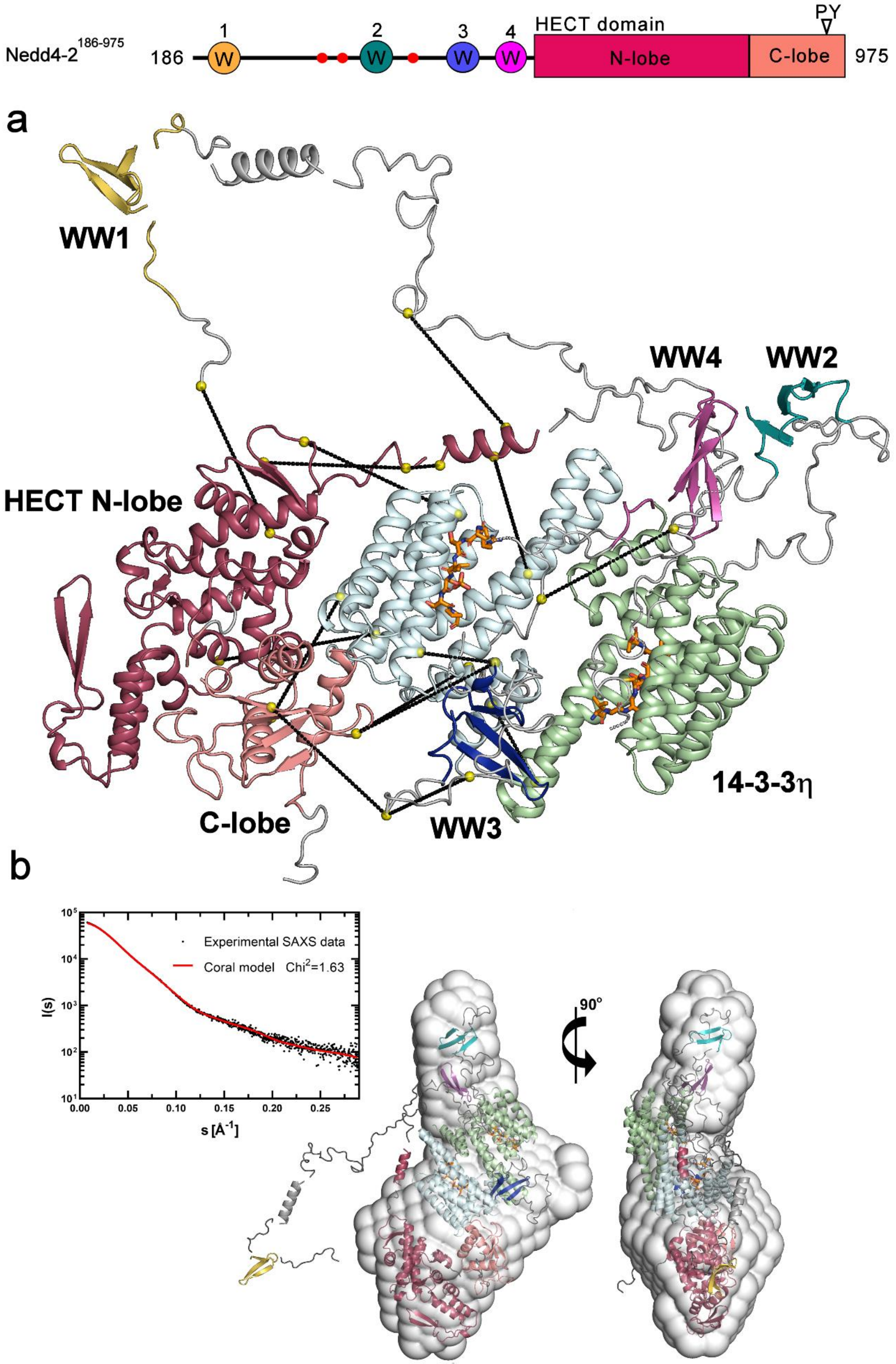
Nedd4-2186-975
186
975
HECT domain
N-lobe
C-lobe
PY
a
WW1
WW4
WW2
HECT N-lobe
C-lobe
WW3
14-3-3η
b
Experimental SAXS data
Coral model Chi2=1.63
I(s)
s [Å-1]
90°

Supplementary Fig. S6: CORAL model of the pNedd4-2$^{186\text{-}975}$:14-3-3η complex. a Best-scoring CORAL model of the Nedd4-2$^{186\text{-}975}$:14-3-3η complex. The 14-3-3η protomers are shown in pale green and pale cyan. In the HECT domain of Nedd4-2$^{186\text{-}975}$, the N-lobe is shown in raspberry and the C-lobe in salmon. WW1, WW2, WW3 and WW4 domains are shown in yellow, teal, blue and magenta, respectively. Structure of phosphorylated peptides in binding grooves solved by X-ray crystallography are represented as orange sticks, colored by elements (PDB ID: 6ZBT and 6ZC9, this work). Flexible linkers between rigid bodies are shown in grey. Cα atoms of crosslinked residues are represented as yellow spheres. **b** Left, scattering curve calculated from the CORAL model of the Nedd4-2$^{186\text{-}975}$:14-3-3η complex (red) compared with experimental SAXS data (black dots) [1]. Right, averaged and filtered *ab initio* molecular envelope of the Nedd4-2$^{186\text{-}975}$:14-3-3η complex (light grey envelope) calculated from the SAXS scattering curve using the program DAMMIF [2]. The averaged and filtered molecular envelope (shown in light grey) was aligned with the CORAL model using the program SUPCOMB [3].

Supplementary Tables:

Supplementary Table S1.
Intramolecular distance constraints of Nedd4-2$^{186\text{-}975}$ derived from the crosslinking experiments with DSG

	Crosslinked peptides		Crosslinked residues		Observed mass	Error (ppm)
#	Nedd4-2	Nedd4-2	Nedd4-2	Nedd4-2		
1.	472-506	472-506	Y485	T495	4071.97	0.4
2.	522-529	507-521	T522	K521	2827.38	0.05
3.	738-742	743-761	K742	T745	2923.34	0.05
4.	596-600	254-301	K598	T275	5748.77	0.38
5.	530-537	538-557	K531	K539	3329.71	-0.1
6.	395-398	254-301	K395	T275	5530.67	0.08
7.	573-580	564-572	T574	K572	2264.06	-0.5
8.	396-400	820-841	K398	K822	3188.70	-0.2
9.	657-665	648-656	Y660	S656	2183.06	0.14
10.	596-600	538-557	K600	S538	2981.50	0.11
11.	494-506	472-493	T495	S493	4089.98	0.75
12.	898-907	871-897	T903	K894	4558.36	-0.1
13.	716-725	726-737	K725	K737	2609.43	0.08
14.	396-400	908-938	K398	S932	4104.03	0.04

Nedd4-2 regions: WW1: 193-226, WW2: 385-418, WW3: 497-530, WW4: 548-581, HECT N-lobe: 595-857, HECT C-lobe: 858-974.

Supplementary Table S2

Intramolecular distance constraints of the Nedd4-2$^{186\text{-}975}$ complex with 14-3-3η derived from the crosslinking experiments with DSG

Crosslink	Crosslinked peptides		Crosslinked residues		Observed	Error
#	Nedd4-2	Nedd4-2	Nedd4-2	Nedd4-2	mass	(ppm)
1.	472-506[a]	472-506	Y485	K493	4071.97	0.08
2.	522-529	507-521	T522	K521	2827.38	-0.3
3.	538-557	410-442	S538	S428	5911.9	0.44
4.	738-742	743-761	K742	T745	2923.34	-0.1
5.	607-617	607-617	K607	K609	1375.81	0.48
6.	596-600	254-301	K598	T275	5748.78	0.78
7.	573-580	564-572	T574	K572	2264.06	-0.4
8.	657-665	648-656	Y660	K656	2183.07	0.21
9.	494-506	472-493	K495	K493	4089.98	0.05
10.	716-725	726-737	K725	Y736	2609.43	-0.1
11.	946-958	959-975	Y951	K960	4663.17	0.18
12.	898-907	871-897	T903	K885	4558.36	-0.1
13.	530-537	908-938	K531	K935	4585.32	0.11

[a]Nedd4-2 regions: WW1: 193-226, WW2: 385-418, WW3: 497-530, WW4: 548-581, HECT N-lobe: 595-857, HECT C-lobe: 858-974.

Supplementary Table S3

Quantitative intramolecular distance constraints of the pNedd4-2$^{186\text{-}975}$ complex with 14-3-3η derived from the crosslinking experiments with 12C DSA/13C DSA.

Crosslink #	Crosslinked peptides		Crosslinked residues		Average ratio pNedd4-2/ pNedd4-2:14-3-3 complex[a]	SD
	Nedd4-2	Nedd4-2	Nedd4-2	Nedd4-2	%	
1.	530-537	606-610	K531	K607	150	30
2.	610-617	618-634	K610	K621	60	10
3.	186-202	625-640	H186	K639	50	10
4.	472-493	865-885	K489	K870	70	10
5.	494-529	530-537	K521	K531	100	0
6.	472-493	871-895	K489	K885	130	10

[a] Representation (%) of the individual crosslink isoform as a ratio between the abundance of 12C DSA (pNedd4-2$^{186\text{-}975}$) and 13C DSA (pNedd4-2$^{186\text{-}975}$:14-3-3η).

Supplementary Table S4
Intermolecular distance constraints of pNedd4-2$^{186\text{-}975}$ complex with 14-3-3η derived from the crosslinking experiments

Crosslink #	Crosslinker	Crosslinked peptides		Crosslinked residues		Observed mass	Error (ppm)
		Nedd4-2	14-3-3η	Nedd4-2	14-3-3		
1.	DSG	599-605	58-61	K600	S58	1553.75	0.23
2.	DSG	530-537	199-227	K531	K217	4451.23	0.05
3.	DSG	522-529	62-78	T522	K69	2827.38	0.87
4.	DSG	886-895	156-172	K894	K162	3185.64	-0.05
5.	DSG	737-742	111-123	K737	K120	2475.10	0.21
6.	DSG	918-935	21-49	K935	K28	5266.49	0.52
7.	DSS	648-656	143-154	K656	K143	2585.26	-0.73

Nedd4-2 regions: WW1: 193-226, WW2: 385-418, WW3: 497-530, WW4: 548-581, HECT N-lobe: 595-857, HECT C-lobe: 858-974.

Supplementary Table S5.

Oligonucleotide sequences for Nedd4-2$^{186-975}$ in pST39 and Nedd4-2$^{335-455}$ in pRSFDuet-1.

**Variant**	**Primer**	**DNA-Sequence (5'-3')**
186-975	pST39-fw	AGTC TCTAGA AAT AAT TTT GTT TAA CTT TAA GAA GGA GAT ATA CAT ATG CAC CAA GAG GAA CTT CCT CC
186-975	pST39-rev	CCG CTG GTA CCC TAG TGG TGA TGA TGG TGA TGG CTG CTG TTA TCC ACC CCT TCA AAT CC
335-455	pHGT2_fw	TGCATGCCATGGGATCCTCAAGGTTGAGG
335-455	pHGT2_rev	GTTGTCCTTTGCGGCCGCCTAAGATAAAGTTACTGTTGG
335-455	S342A-fw	GGTTGAGGTCATGCGCTGTCACCGACGCAG
335-455	S342A-rev	CTGCGTCGGTGACAGCGCATGACCTCAACC
335-455	T367A-fw	CGCGTTCATCAGCTGTCACGGGTGGTG
335-455	T367A-rev	CACCACCCGTGACAGCTGATGAACGCG
335-455	S448A-fw	GGCCTCGTAGCCTCGCCTCGCCAACAGTAAC
335-455	S448A-rev	GTTACTGTTGGCGAGGCGAGGCTACGAGGCC

Supplementary References

1. Petoukhov, M.V. et al. New developments in the ATSAS program package for small-angle scattering data analysis. *Journal of Applied Crystallography* **45**, 342-350 (2012).
2. Franke, D. & Svergun, D.I. DAMMIF, a program for rapid ab-initio shape determination in small-angle scattering. *Journal of Applied Crystallography* **42**, 342-346 (2009).
3. Kozin, M.B. & Svergun, D.I. Automated matching of high- and low-resolution structural models. *Journal of Applied Crystallography* **34**, 33-41 (2001).
