# Supplementary material for: 14-3-3-protein regulates Nedd4-2 by modulating interactions between HECT and WW domains
Source: Commun Biol. 2021 Jul 22;4:899. doi: 10.1038/s42003-021-02419-0 (PMC8298602; doi:10.1038/s42003-021-02419-0)
Supplement: Supplementary file 2 — Reporting Summary [file 42003_2021_2419_MOESM2_ESM.pdf]

nature research

Corresponding author(s): Veronika Obsilova

Last updated by author(s): Jun 10, 2021

Reporting Summary

Nature Research wishes to improve the reproducibility of the work that we publish. This form provides structure for consistency and transparency in reporting. For further information on Nature Research policies, see our Editorial Policies and the Editorial Policy Checklist.

Statistics

For all statistical analyses, confirm that the following items are present in the figure legend, table legend, main text, or Methods section.

n/a	Confirmed	
☐	☒	The exact sample size (*n*) for each experimental group/condition, given as a discrete number and unit of measurement
☐	☒	A statement on whether measurements were taken from distinct samples or whether the same sample was measured repeatedly
☐	☒	The statistical test(s) used AND whether they are one- or two-sided<br>*Only common tests should be described solely by name; describe more complex techniques in the Methods section.*
☒	☐	A description of all covariates tested
☒	☐	A description of any assumptions or corrections, such as tests of normality and adjustment for multiple comparisons
☐	☒	A full description of the statistical parameters including central tendency (e.g. means) or other basic estimates (e.g. regression coefficient) AND variation (e.g. standard deviation) or associated estimates of uncertainty (e.g. confidence intervals)
☒	☐	For null hypothesis testing, the test statistic (e.g. *F*, *t*, *r*) with confidence intervals, effect sizes, degrees of freedom and *P* value noted<br>*Give P values as exact values whenever suitable.*
☒	☐	For Bayesian analysis, information on the choice of priors and Markov chain Monte Carlo settings
☒	☐	For hierarchical and complex designs, identification of the appropriate level for tests and full reporting of outcomes
☒	☐	Estimates of effect sizes (e.g. Cohen's *d*, Pearson's *r*), indicating how they were calculated

Our web collection on statistics for biologists contains articles on many of the points above.

Software and code

Policy information about availability of computer code

Data collection	Data for crystals were collected at beamline 14.1 of the BESSY synchrotron and at D8 Venture system (Bruker, MA, USA) at BIOCEV. SAXS data were collected on the European Molecular Biology Laboratory (EMBL) P12 beamline, at the storage ring PETRA III (Deutsches Elektronen Synchrotron (DESY), Hamburg, Germany).
Data analysis	Data were analyzed using: GraphPad Prism version 8.0.1 for Windows, Image Lab, SEDPHAT, SEDNTERP software, XDS and XDSAPP packages, MOLREP, PHENIX, PyMOL, xiVIEW, CORAL and ATSAS 3.0.2 software package, StavroX and Links software as indicated in the Methods section.

For manuscripts utilizing custom algorithms or software that are central to the research but not yet described in published literature, software must be made available to editors and reviewers. We strongly encourage code deposition in a community repository (e.g. GitHub). See the Nature Research guidelines for submitting code & software for further information.

Data

Policy information about availability of data

All manuscripts must include a data availability statement. This statement should provide the following information, where applicable:

- Accession codes, unique identifiers, or web links for publicly available datasets
- A list of figures that have associated raw data
- A description of any restrictions on data availability

The atomic coordinates and structure factors were deposited in the Protein Data Bank (www.wwpdb.org) under accession codes 6ZBT, 6ZC9 and 7NMZ.
All data are available in the Supplementary Data 1 file.
The authors declare that data supporting the findings of this study are available within the paper and its supplementary information files.

Field-specific reporting

Please select the one below that is the best fit for your research. If you are not sure, read the appropriate sections before making your selection.

☒ Life sciences ☐ Behavioural & social sciences ☐ Ecological, evolutionary & environmental sciences

For a reference copy of the document with all sections, see nature.com/documents/nr-reporting-summary-flat.pdf

Life sciences study design

All studies must disclose on these points even when the disclosure is negative.

Sample size	Sample sizes (n) for each experimental group are indicated in the Fig. legends.
Data exclusions	No data were excluded.
Replication	At least three independent experiments were performed for each experimental group.
Randomization	Not relevant.
Blinding	Not relevant.

Reporting for specific materials, systems and methods

We require information from authors about some types of materials, experimental systems and methods used in many studies. Here, indicate whether each material, system or method listed is relevant to your study. If you are not sure if a list item applies to your research, read the appropriate section before selecting a response.

Materials & experimental systems

n/a	Involved in the study	
☐	☒	Antibodies
☒	☐	Eukaryotic cell lines
☒	☐	Palaeontology and archaeology
☒	☐	Animals and other organisms
☒	☐	Human research participants
☒	☐	Clinical data
☒	☐	Dual use research of concern

Methods

n/a	Involved in the study	
☒	☐	ChIP-seq
☒	☐	Flow cytometry
☒	☐	MRI-based neuroimaging

Antibodies

Antibodies used	anti-rabbit IgG, HRP-linked Antibody (Cell Signaling Technology, catalogue no: 7074S)<br>Rabbit anti-Ub polyclonal antibody (Enzo, catalogue no.: ADI-SPA-200-D)
Validation	HRP-linked Antibody (Cell Signaling Technology) were used to visualize the transferred polyubiquitin chains. Chemiluminescence was induced by ECL and detected using Fusion Solo S (Vilber). The detected bands of E3-Ub were quantitated using the Image Lab software (Bio-Rad) (Figure S1).
