# Supplementary material for: 14-3-3-protein regulates Nedd4-2 by modulating interactions between HECT and WW domains
Source: Commun Biol. 2021 Jul 22;4:899. doi: 10.1038/s42003-021-02419-0 (PMC8298602; doi:10.1038/s42003-021-02419-0)
Supplement: Supplementary file 3 — Description of Additional Supplementary Files [file 42003_2021_2419_MOESM3_ESM.pdf]

Description of Additional Supplementary Files

File name: Supplementary Data 1

Description: Raw data for Figures 2-4, 7 and Supplementary Figures S1-S6.
